# Stakeholder-Driven Methods Can Enhance Care Delivery for Nursing Home Residents With Dementia

**DOI:** 10.1093/geroni/igab046.2095

**Published:** 2021-12-17

**Authors:** Natalie Leland, Felicia Chew, Jenny Martínez

## Abstract

The ongoing COVID-19 pandemic has underscored the need to optimize care for one of the most affected sectors: older adults in nursing homes and more specifically highly vulnerable populations such as residents with dementia. Research developed in collaboration with stakeholders can optimize impact, relevance, and trustworthiness of study findings thereby informing advances in care. Yet, evidence on stakeholder driven research for enhancing dementia care is limited. This symposium will provide examples of stakeholder-driven research questions that were addressed with stakeholder engagement. First, we will present current evidence about the perspectives of caregivers, including those from communities of color. The second presentation will discuss the perspective of clinical training stakeholders responsible for supporting system-wide clinical program implementation and their experiences with early and later adopter nursing homes within the context of a clinical trial. The third presentation will address the perspective of policy makers and payers via the effect of state-mandated dementia training on resident outcomes. The fourth and final present the findings from a study that examined how nursing home stakeholders responded to a payor requirements for pharmacy services and the relationship between that response and patient outcomes. We will conclude the session with a discussion of stakeholder-engagement methods and recommendations for future nursing home research, which champions stakeholder collaboration.

